# The Eulogium of Dr. Dake

**Published:** 1895-03

**Authors:** G. J. Waggoner

**Affiliations:** Larned, Kan.


### THE EULOGIUM OF DR. DAKE.

LARNED, KAN., Jan. 12th, 1895.

EDITOR OF THE HOMŒOPATHIC PHYSICIAN:

I wish to enter my earnest protest against the publication, in THE HOMŒOPATHIC PHYSICIAN, of lengthy eulogiums on such doctors as J. P. Dake. It is as well known to the editor of said journal as to hundreds of homœopathic physicians that Dr. Dake was an avowed heretic in Homœopathy, a traitor to Hahnemannian methods, and a pervertionist of our materia medica, as THE HOMŒOPATHIC PHYSICIAN has often declared and set forth. No one could object to a simple obituary notice, but when it comes to six or eight pages of fulsome adulation of an arch-traitor and a perversionist of those principles that we hold dearer than life, it is time to call a halt.

Very sincerely,

G. J. WAGGONER.

